# Inhibition of c‐MET increases the antitumour activity of PARP inhibitors in gastric cancer models

**DOI:** 10.1111/jcmm.15655

**Published:** 2020-07-20

**Authors:** Evangelos Koustas, Michalis V. Karamouzis, Panagiotis Sarantis, Dimitrios Schizas, Athanasios G. Papavassiliou

**Affiliations:** ^1^ Molecular Oncology Unit Department of Biological Chemistry Medical School National and Kapodistrian University of Athens Athens Greece; ^2^ First Department of Internal Medicine, 'Laiko' General Hospital Medical School National and Kapodistrian University of Athens Athens Greece; ^3^ First Department of Surgery Medical School National and Kapodistrian University of Athens Athens Greece

**Keywords:** BRCA1, BRCA2, c‐Met inhibitor, gastric cancer, PARP inhibitor

## Abstract

Gastric cancer is the fifth most common malignancy and the third leading cause of cancer‐related death worldwide. Activation of c‐MET increases tumour cell survival through the initiation of the DNA damage repair pathway. PARP is an essential key in the DNA damage repair pathway. The primary role of PARP is to detect and initiate an immediate cellular response to single‐strand DNA breaks. Tumours suppressor genes such as BRCA1/2 are closely associated with the DNA repair pathway. In BRCA1/2 mutations or deficiency status, cells are more likely to develop additional genetic alterations and chromosomal instability and can lead to cancer. In this study, we investigate the role of c‐MET and PARP inhibition in a gastric cancer model. We exploited functional in vitro and in vivo experiments to assess the antitumour potential of co‐inhibition of c‐MET (SU11274) and PARP (NU1025). This leads to a reduction of gastric cancer cells viability, especially after knockdown of BRCA1/2 through apoptosis and induction of γ‐Η2ΑΧ. Moreover, in AGS xenograft models, the combinatorial treatment of NU1025 plus SU11274 reduced tumour growth and triggers apoptosis. Collectively, our data may represent a new therapeutic approach for GC thought co‐inhibition of c‐MET and PARP, especially for patients with BRCA1/2 deficiency tumours.

## 1 INTRODUCTION

Gastric cancer is the 5th most common malignancy and the third leading cause of cancer‐related death worldwide.^1^, ^2^ Several studies identified c‐MET as a major regulator of tumorigenesis in GC through the initiation of the DNA damage repair pathway.^3^

Although mutations of the MET gene are not common in GC,^4^ MET protein overexpression rates in 50% of advanced gastric cancers^5^ and accordingly, MET gene amplification rates vary from 4%‐10% of gastric tumour patients.^6^, ^7^ In the HS746T GC cell line, a mutation in exon 14 of c‐MET triggers the deletion of the juxtamembrane domain.^8^, ^9^ Thus, several studies already use antibodies such as rilotumumab or onartuzumab to inhibit HGF/MET in different types of cancer.^10^, ^11^

Several studies have shown that 8% of GC tumours are characterized by MSI‐H phenotype, which results in an insufficient DNA mismatch repair^12^, ^13^ and higher resistance to chemotherapy and radiotherapy.^14^ Thus, inhibition of DNA damage response (DDR) mechanisms, especially with PARP1 depletion in BRCA1/2‐deficient models, may decrease the survival of cancer cells and promote a more effective antitumour therapy.^15^

One crucial role of PARP is assisting in the repair of single‐strand DNA breaks. As a result, PARP inhibition leads to DNA double‐strand breaks (DSBs) that are the most deleterious form of DNA damage.^16^ Clinical trials (NCT01063517 and GOLD, NCT01924533, respectively) use agents that focus on this DNA repair pathway mechanism. In more detail, phase II/III clinical studies use PARP inhibitor in the chemotherapeutic scheme with paclitaxel. This co‐treatment showed a beneficial effect on the survival rating of patients.^15^, ^16^, ^17^, ^18^ In light of the results from clinical studies, PARP inhibition in GC patients tries to improve our understanding of DSBs repair pathways and find new and more reliable predictive markers for this kind of cancer.^19^, ^20^

BRCA1/2 proteins are necessary for the HR progression as the cells are susceptible to PARP inhibition when the BRCA1/2 protein is deficient.^21^, ^22^ Many studies of BRCA1/2 mutations and GC are indirect and do not show the rate of BRCA1/2 mutations in patients with GC.^23^ However, the link between BRCA1/2 mutation and increased risk of GC was verified in previous studies for families with hereditary breast and ovarian cancer.^24^, ^25^, ^26^ In an analysis done in Israel, 5.7% of patients were detected with GC with specific BRCA2 mutations.^27^ Zhang et al showed that loss of BRCA1 occurred in 21.4% of patients with GC. Patients with BRCA1 loss have reduced life expectancy due to higher tumour grade and advanced clinical stage.^28^ Mutations in BRCA1/2 mutations increase the risk of developing CG around sixfold, especially between first‐degree relatives.^29^

It has been shown that c‐MET stimulation is necessary to develop resistance to the DNA damaging agent.^30^, ^31^ Another study reports that inhibition of MET, in MET‐overexpressing GC model, causes damage to the DNA, resulting in premature ageing.^32^, ^33^

In the current study, we try to explore the combination of c‐met and PARP inhibition in GC cell lines models (AGS and HS746T). In more detail, co‐treatment of GC cell lines with NU1025 and SU11274 (PARP and c‐MET inhibitor, respectively) decreased cell viability through induction of apoptotic cell death in BRCA1/2 deficiency manner. Furthermore, in vivo experiment in AGS xenograft mouse model, co‐inhibition of c‐MET and PARP decreases tumour volume mass. Collectively, we proposed that co‐treatment of PARP and c‐MET inhibitors had a beneficial effect in the BRCA1/2 deficiency GC model and are a putative therapeutic approach for GC patients.

## 2 MATERIALS AND METHODS

### 2.1 Inhibitors and drugs

The c‐MET inhibitor SU11274 (#S9820) and PARP inhibitor NU1025 (#N7287) were obtained from Sigma‐Aldrich. Both inhibitors were dissolved in DMSO and stored at − 80°C.

### 2.2 Cell culture

Hs746T and AGS GC cell lines were obtained from American Type Culture Collection (ATCC) and American Type European Collection of Authenticated Cell Cultures (ECACC).

All cell lines used in this study were grown in RPMI Medium 1640—GlutaMAX™ (#61870‐010 Life Technologies Carlsbad) supplemented with 10% foetal bovine serum (FBS), penicillin and streptomycin antibiotics (all from Invitrogen). Cells were maintained at 37°C in a humidified incubator containing 5% CO_2_. The experiments were done with the approval of the Ethics Committee of our University.

### 2.3 siRNA and transfection

For the siRNA transfection experiments, we use siRNA for stable knockdown of c‐MET (sc‐35924), BRCA1 (sc‐29219) and BRCA2 (sc‐29825) and control (sc‐37007) from Santa Cruz Biotechnology and Lipofectamine 3000 (#L3000‐15 Invitrogen Corp.).

### 2.4 Cell viability assay

Cell growth and viability were confirmed by MTT assay. Approximately 3,000 cells were placed in a 96‐well plate with 200 μL culture medium. At the end of treatment time, cells were incubated for 4 hours with 0.8 mg/mL of MTT, dissolved in a serum‐free medium followed by DMSO (1 mL) and gentle shaking for 10 minutes to achieve the complete dissolution. Finally, the absorbance was measured at 560 nm using the microplate spectrophotometer system (SpectraMax 190‐Molecular Devices). Results are presented as percentage of the control values.

### 2.5 Western blot assay

As described in detail previously,^34^ RIPA buffer (#9806 Cell Signaling Technology) is used to prepare whole‐cell lysates. A total of 25 μg of protein (concentration was determined using the Bradford method) was resolved on SDS‐PAGE and transferred to nitrocellulose membrane (Whatman, Scheicher & Schuell). Antibodies were used against: BRCA1 (#MCA5946GT) was purchased from Bio‐Rad, BRCA2 (sc‐295185), actin (sc‐8035) and histone H2AX (#sc‐517336) were purchased from Santa Cruz Biotechnology, and PARP‐1 (#9542), cleaved caspase‐3 (#9661) and c‐Met (#8198) from Cell Signaling Technology. The normalization of protein levels is against actin. The experiments represent three independent experiments, and the standard deviation is presented. The protein band intensities were measured by ImageJ.

### 2.6 Two‐dimensional culture and confocal microscopy

For the 2D culture experiments, cells (5000 cells/well) were grown on coverslips in 24‐well plates in medium, at 37°C. After knockdown of BRCA1/2 and treatment with NU1025 or SU11274, alone or in combination, for 24 hours cells were fixed in 4% paraformaldehyde, then permeabilized and then blocked with 0.5% BSA/PBS‐5% 22 Triton X‐100. Next, cells were treated with the primary H2AX antibody and then incubated with an antimouse fluorescence‐labelled secondary antibody (#20014). Cells were examined using an Olympus FV1000 confocal microscope with an Olympus digital camera. The nuclei were stained with Dapi No. 33 342.

### 2.7 Wound healing assay

HS746T GC cell line (40 000 cells/well) was grown in a 12‐well plate and after knockdown of BRCA1/2 with siRNA transfection, cells were incubated with 5 μmol/L NU1025 and/or SU11274, alone or in combination, for 24 hours. At day 0, we formed the wound with a yellow pipette tip. After 24 hours of incubation, cells were photographed utilizing computer‐assisted microscopy. We measured the gap distance of the wound on day 0 and after 24 hours using Image‐Pro Plus software.

### 2.8 Clonogenic cell survival assay

HS746T GC cell lines were plated into a 6‐well plate. After knockdown of BRCA1/2 with siRNA transfection, cells were incubated with 5 μmol/L NU1025 and/or SU11274, alone or in combination, for 14 hours. We renew the inhibitors every 2 days. Following 14 days of incubation, colonies were fixed with methanol: acetic acid (3:1) solution and stained with haematoxylin. Cells were subsequently washed with PBS, dried and imaged.

### 2.9 Mouse xenograft models

For the in vivo experiments, we used NSG MICE. All scid mice, housed in micro isolator cages, were used between 6 and 8 weeks of age. All procedures were carried out in accordance with the guidelines for animal experimentation following the European Union of the National and Kapodistrian University of Athens, Medical School Bioethics Committee in agreement with the European Union (approval no. 3233/26‐06‐2018). For injection of cell suspensions, we used 1‐5 million cells in 100 μL into the right flank of each mouse and allowed to grow for approximately 3‐4 weeks to reach a tumour size of 100 mm3. The mice were randomly divided into groups (n = 5 per group) for each treatment, control, NU1025 (1 mg/mouse), SU11274 (1 mg/mouse) and NU1025 + SU11274. Inhibitors were injected intraperitoneally every 4 days. The mice were killed, and solid tumours were measured and excised after 20 days of treatment. The tumour volume was calculated using the following formula: 1/2(length × width2).

### 2.10 Animal care and use statement

The animal protocol was designed to minimize pain or discomfort to the animals. The animals were acclimatized to laboratory conditions (23°C, 12‐h/12‐h light/dark, 50% humidity, and libitum access to food and water) for 2 weeks prior to experimentation. Intragastric gavage administration was carried out with conscious animals, using straight gavage needles appropriate for the animal size (15‐17 g body weight: 22 gauge, 1‐inch length, 1.25 mm ball diameter). All animals were killed by barbiturate overdose (intravenous injection, 150 mg/kg pentobarbital sodium) for tissue collection.

### 2.11 Statistical analysis

The results are representative of three independent experiments and expressed as mean values ± SD (standard deviation). For sample size, we used G*Power software version 3.1. For the calculation of tumour volume, we used Microsoft Excel 10. The results were evaluated by t test. Error bars indicate ± SD. *P < .05, **P < .005, ***<0.0005.

## 3 RESULTS

### 3.1 Steady levels in primary gastric cancer cell lines

**FIGURE 1:**
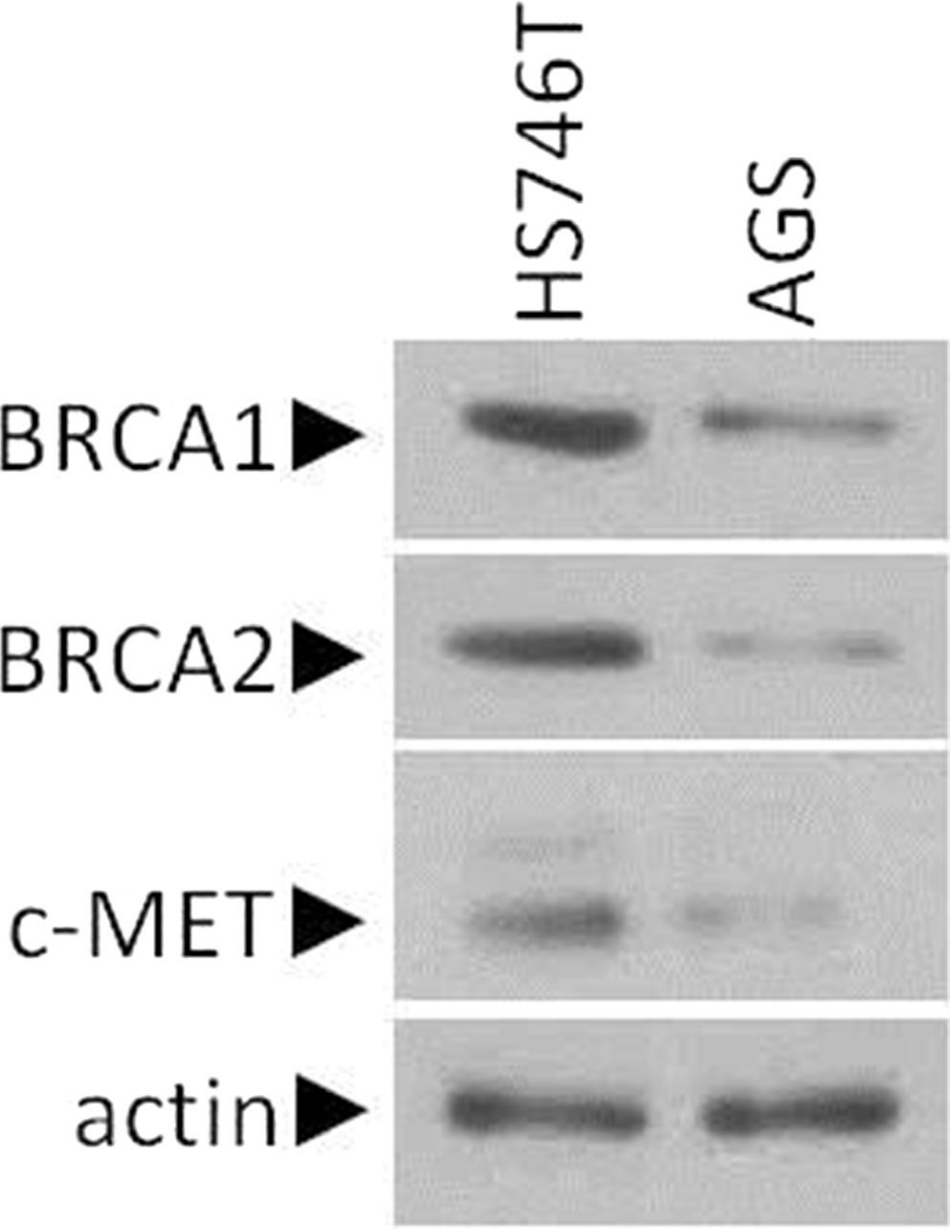
Steady‐state levels of gastric cancer cell lines. Using Western blot assay, steady protein levels of BRCA1, BRCA2 and c‐MET are analysed in primary gastric cancer cell lines HS746T and AGS. Protein levels were normalized against actin

Different gastric cancer cell lines were examined regarding their protein levels of BRCA1, BRCA2 and c‐MET using Western blot analysis. In detail, the protein levels of BRCA1, BRCA2 and c‐MET were decreased in the AGS cell line as compared to HS746T (Figure 1).

### 3.2 The role of c‐MET in PARP inhibition response in GC cell lines

**FIGURE 2:**
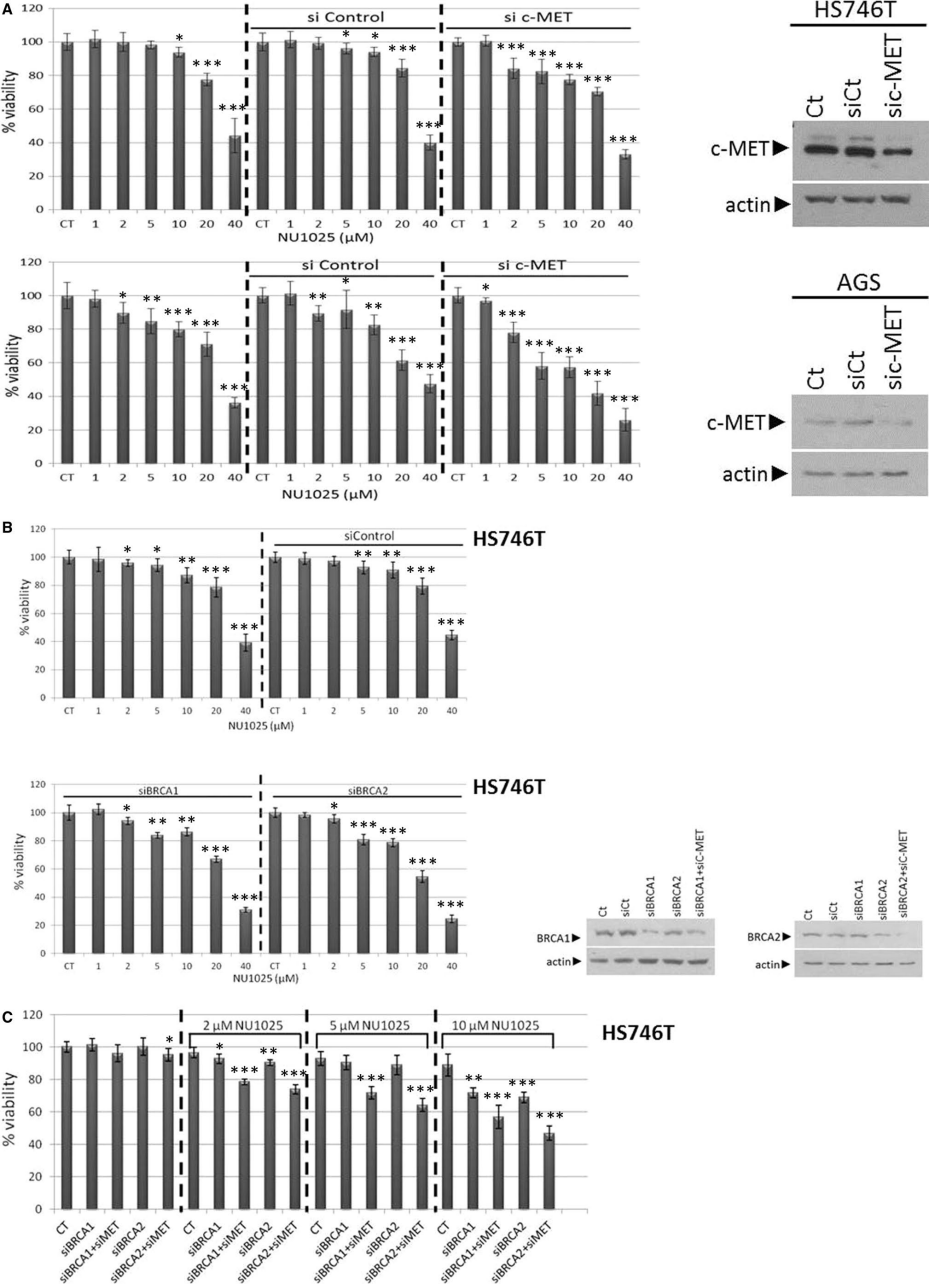
Low levels of c‐MET partially sensitize GC cell lines in PARP inhibition. A, HS746T/AGS cells, control‐siRNA‐Hs746T/AGS cells and si‐c‐MET Hs746T/AGS cells were exposed to increasing doses (0‐40 µmol/L) of NU1025 for 48 h for determination of cell viability (MTT metabolic activity assay). The protein levels of c‐MET expression (by Western blot analysis) revealed down‐regulation of the c‐MET receptor in both cell lines (HS746T and AGS); (B) HS746T cells, control‐siRNA‐Hs746T cells and si‐BRCA1/2 Hs746T cells were exposed to increasing doses (0‐40 µmol/L) of NU1025 for 48 h for determination of cell viability (MTT metabolic activity assay). The protein levels of BRCA1 and BRCA2 expression (by Western blot analysis) revealed down‐regulation of the BRCA1/2 in HS746T cell line; (C) HS746T cells, control‐siRNA‐Hs746T, siBRCA1/2‐Hs746T and siMET/BRCA1/2‐Hs746T cells were cultured with the indicated concentrations of NU1025 (5, 10 and 20 μmol/L) for 48 h for determination of cell viability (MTT metabolic activity assay). Error bars represent SD

We identified the effect of c‐MET activation on cell viability by PARP inhibition (NU1025) in an increasing dose‐dependent manner (0‐40 μmol/L) for 48 hours with MTT assay. We used HS746T and AGS GC cell lines to exhibit high and low protein levels of c‐MET, respectively (Figure 2A). Although we observed that NU1025 strongly reduces cell proliferation of AGS and HS746T cells in a higher concentration of 20 μmol/L, HS746T cell line appears to be more resistant to lower concentrations of NU1025 than AGS cells (Figure 2A). In 2‐10 μmol/L of NU1025, the reduction of cell proliferation in AGS cell lines is almost double than HS746T after the silence of c‐MET (Figure 2A). Subsequently, an experiment with knockdown of c‐MET expression with siRNA was performed in both cell lines (HS746T and AGS; Figure 2A). With Western blot analysis, we tested the effect of siRNA‐mediated knockdown of c‐MET in GC cell lines (Figure 2A). The silence of c‐MET sensitizes Hs746T and AGS cells to PARP inhibition (NU1025). These results were shown in the per cent of cell viability, where the reduction of cell viability was monitored (Figure 2A).

### 3.3 The impact of BRCA and c‐MET on PARP inhibition in GC cells

In order to identify whether the expression of BRCA has differential drug sensitivity, we continue with the silence of BRCA1/2 by siRNA in the HS746T cell line (Figure 2B). Then, HS746T cells were treated with increasing concentrations (0‐40 μmol/L) of PARP inhibitor (NU1025) for 48 hours. We evaluate that the silence of BRCA1/2 increases the sensitivity of cell after 5 μmol/L of NU1025 (Figure 2B). Effectively silence of BRCA1/2 was confirmed by the reduction of protein levels of BRCA1 or BRCA2 by Western blot analysis (Figure 2B). siRNA of BRCA1 or BRCA2 displayed a slight difference between controls. These data support that BRCA1 or 2 siRNA‐mediated knockdowns affect cell viability of HS746T cell lines.

Additionally, in order to identify the primary mechanism of expression of BRCA impact on c‐MET, we continued with the silence of c‐MET. The knockdown of c‐MET increases the sensitivity of BRCA‐deficient HS746T cells to NU1025 after 48 hours (Figure 2C). BRCA1/2‐deficient Hs746T cells revealed a slightly higher growth‐inhibitory impact in comparison with the BRCA1/2‐proficient cells after 48 hours (Figure 2B).

### 3.4 Inhibition of c‐MET (SU11274) sensitizes and triggers apoptosis in GC cells with BRCA1/2 deficient to PARP inhibition (NU1025)

**FIGURE 3:**
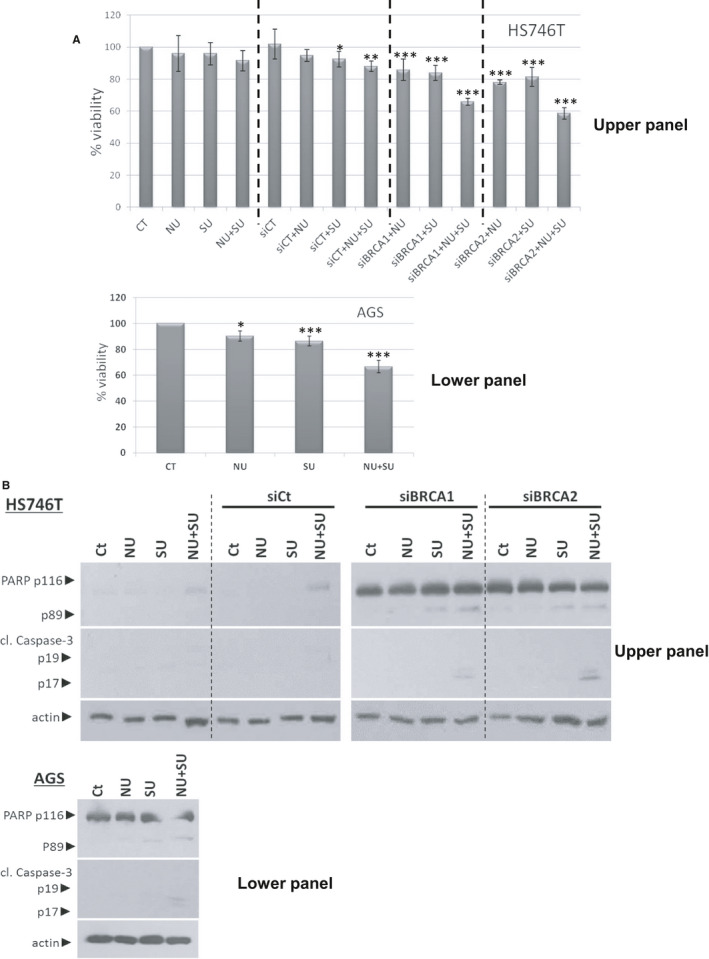
Co‐inhibition of c‐MET (SU11274) and PARP (NU1025) sensitizes GC cells after knockdown BRCA1/2. Knocking down BRCA1 or BRCA2 sensitizes cells to PARP and c‐MET inhibition in HS746T cells expressing low levels of c‐MET (AGS cells, c‐MET knockdown Hs746T cells) to PARP inhibition. A, HS746T cells, control‐siRNA‐Hs746T cells and siBRCA1/2‐Hs746T (upper panel) and AGS (lower panel) cells were exposed to 5 µmol/L of NU1025 and/or 5 µmol/L of SU11274 for 48 h for determination of cell viability (MTT metabolic activity assay). Results are expressed as percentages. Average values of three experiments ± SD are shown; (B) Western blot analysis of PARP and cl.caspase‐3 in Hs746T‐control‐siRNA, siBRCA1/2‐Hs746T (upper panel) and AGS (lower panel) cell lines. Cells were cultured with the indicated drugs (5 μmol/L NU1025, 5 μmol/L SU11274 alone or in combination for 24 h of treatment). Protein levels were normalized against actin

In the light of previous results that silence of c‐MET and/or BRCA1/2 sensitizes AGS and Hs746T cells to NU1025 treatment, we continue with the investigation of an additive effect in co‐inhibition of c‐MET (5 μmol/L SU11274) and PARP (5 μmol/L NU1025) inhibitor in order to further reduce the proliferation of GC cell lines. When Hs746T cells (control and siControl) were treated with SU11274 or NU1025 alone or combined, the additive effect was not observed after 48 hours (Figure 3A upper panel). As a next step, we experimented in order to silence BRCA1 or BRCA2 with siRNA in HS746T cell lines. After successful knockdown of BRCA1 or BRCA2 (as it was identified through Western blot analysis—Figure 3A upper panel), HS746T cells were treated with SU11274 or NU1025 alone or in combination. In a combinatorial scheme, an additive reduction of cell proliferation 34.3% for siBRCA1 and 42% for siBRCA2 was observed after 48 hours (Figure 3A upper panel). Moreover, we observed a substantial reduction of cell viability of AGS cell lines (~34%) when combined 5 μmol/L of SU11274 plus 5 μmol/L NU1025 after 48 hours (Figure 3A lower panel).

In the next step, we investigated the way of reduction in cell viability of GC cell lines HS746T and AGS. We showed that BRCA1 or BRCA2 deficiency in Hs746T cells triggers apoptotic cell death as it was monitored through the detection of PARP‐1 and cleaved caspase‐3 by Western blot analysis when combined 5 μmol/L of SU11274 plus 5 μmol/L NU1025 (Figure 3B upper panel). In addition, apoptotic cell death was observed in AGS cell lines when combined 5 μmol/L of SU11274 plus 5 μmol/L NU1025 compounds for 24 hours (Figure 3B lower panel). It is clear that BRCA1 or BRCA2 protects the cells from c‐MET and/or PARP inhibition as it was examined and identified through MTT assay and detection of apoptotic markers PARP and cl.casapse‐3 through Western blot analysis.

### 3.5 SU11274 plus NU1025 reduces clonogenicity in BRCA1/2‐deficient Hs746T cells

To identify the impact of BRCA1 and BRCA2 on basic cell properties, we performed a wound migration and clonogenic assay in the HS746T cell line. After effective knockdown of BRCA1 and 2, we treated HS746T cells with 5 μmol/L of SU11274 and 5 μmol/L NU1025 alone or in combination for 24 hours. As it was evaluated by migration assay, NU1025 plus SU11274 in siBRCA1/2‐Hs746T cells did not affect the metastatic potential of this cell line (Figure S1A).

We continued with the clonogenic survival assay to evaluate the effect of NU1025 and SU11274 on colony formation of the HS746T cell line. 5 μmol/L of SU11274 plus 5 μmol/L NU1025 in siBRCA1/2‐Hs746T cells strongly suppressed clonogenicity of HS746T cell line (Figure S1B).

### 3.6 Co‐inhibition of c‐MET and PARP enhances the levels of γ‐H2AX and DNA damages

**FIGURE 4:**
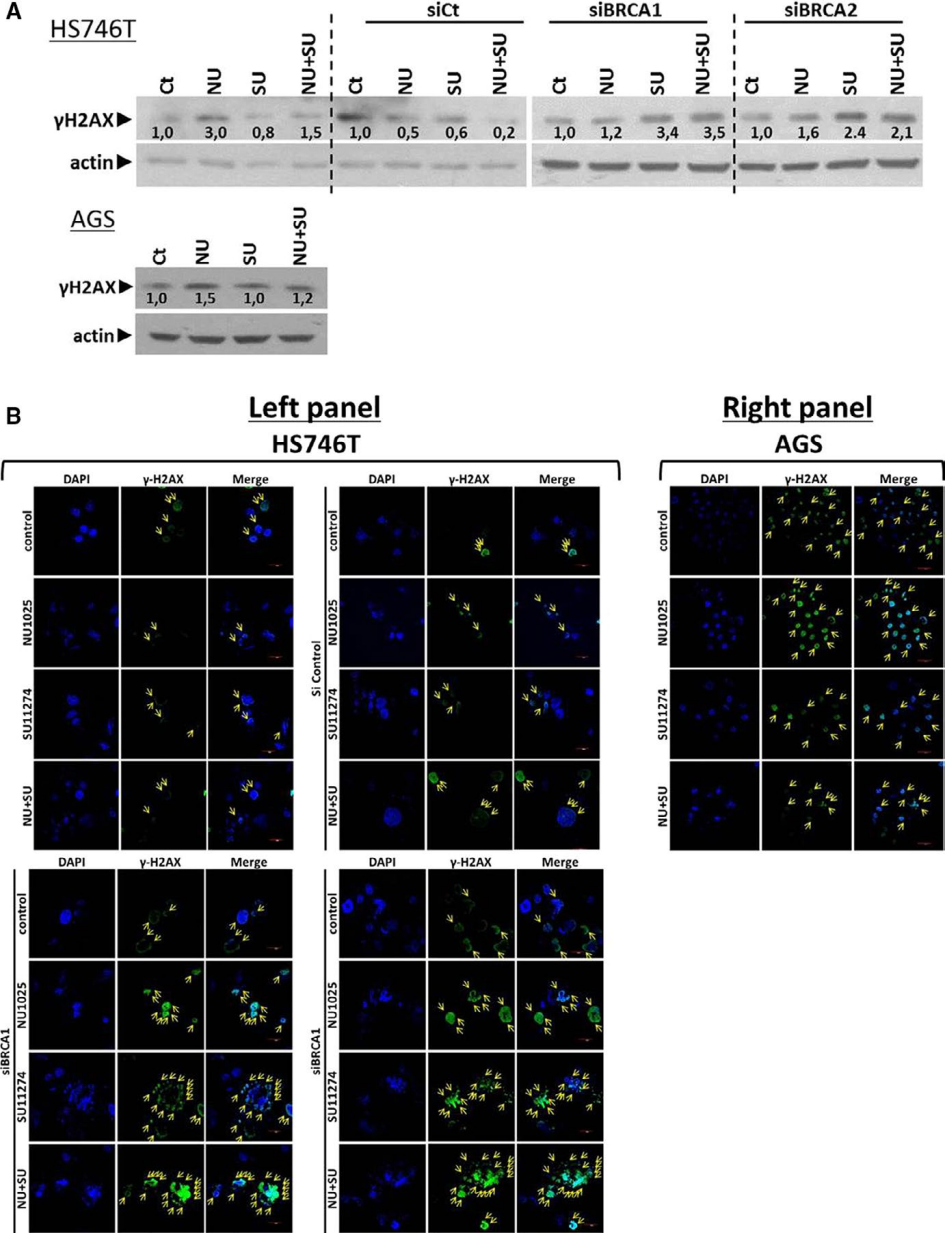
NU1025 plus SU11274 increases DNA damage in GC cell line. A, Western blot analysis of γ‐H2AX in Hs746T‐control‐siRNA, siBRCA1/2‐Hs746T and AGS cell lines. Cells were cultured with the indicated drugs (5 μmol/L NU1025, 5 μmol/L SU11274 alone or in combination after 24 h of treatment). Protein levels were normalized against actin; (B) confocal microscope images of two‐dimensional culture of HS746T (left panel) and AGS (right panel) cell lines. Hs746T‐control‐siRNA, siBRCA1/2‐Hs746T and AGS cells were cultured with the indicated drugs and concentrations for 24 h. Representative images of Hs746T and AGS nuclei (DAPI‐blue staining) and of γ‐H2AX (green) are shown in the figure

Our results showed that NU1025 plus SU11274 in BRCA1/2 deficiency cell line HS746T and AGS cell line reduces cell viability through apoptotic cell death. In order to examine DSBs and genotoxic treatments in chromatin, we examined the protein levels of γ‐H2AX in siBRCA1/2‐HS746T and AGS cell lines under the effect of 5 μmol/L of SU11274 plus 5 μmol/L NU1025 for 24 hours. The Western blotting analysis identified the elevated protein levels of γ‐H2AX in NU1025 plus SU11274 in both cell lines (Figure 4A). These findings evaluate that silence of BRCA1/2 and treatments with 5 μmol/L SU11274 plus 5 μmol/L NU1025 activates the expression of γ‐H2AX and initiates DNA damage in HS746T cell line. In the AGS cell line, the combinatorial scheme of 5 μmol/L SU11274 and 5 μmol/L NU1025 for 24 hours slightly increased the expression of γ‐H2AX (Figure 4A). Furthermore, we noticed that the proportion of γ‐H2AX‐positive cells (green fluorescence and yellow arrows show the high density of γ‐H2AX) was increased after combined treatment (5 μmol/L SU11274 plus 5 μmol/L NU1025 for 24 hours) in HS746T cell line and less in AGS cell line as it was confirmed by immunofluorescence microscopy (Figure 4B left and right panel).

### 3.7 The NU1025 plus SU11274 combinatorial treatment reduces the tumour and triggers apoptotic cell death growth in AGS xenograft models

**FIGURE 5:**
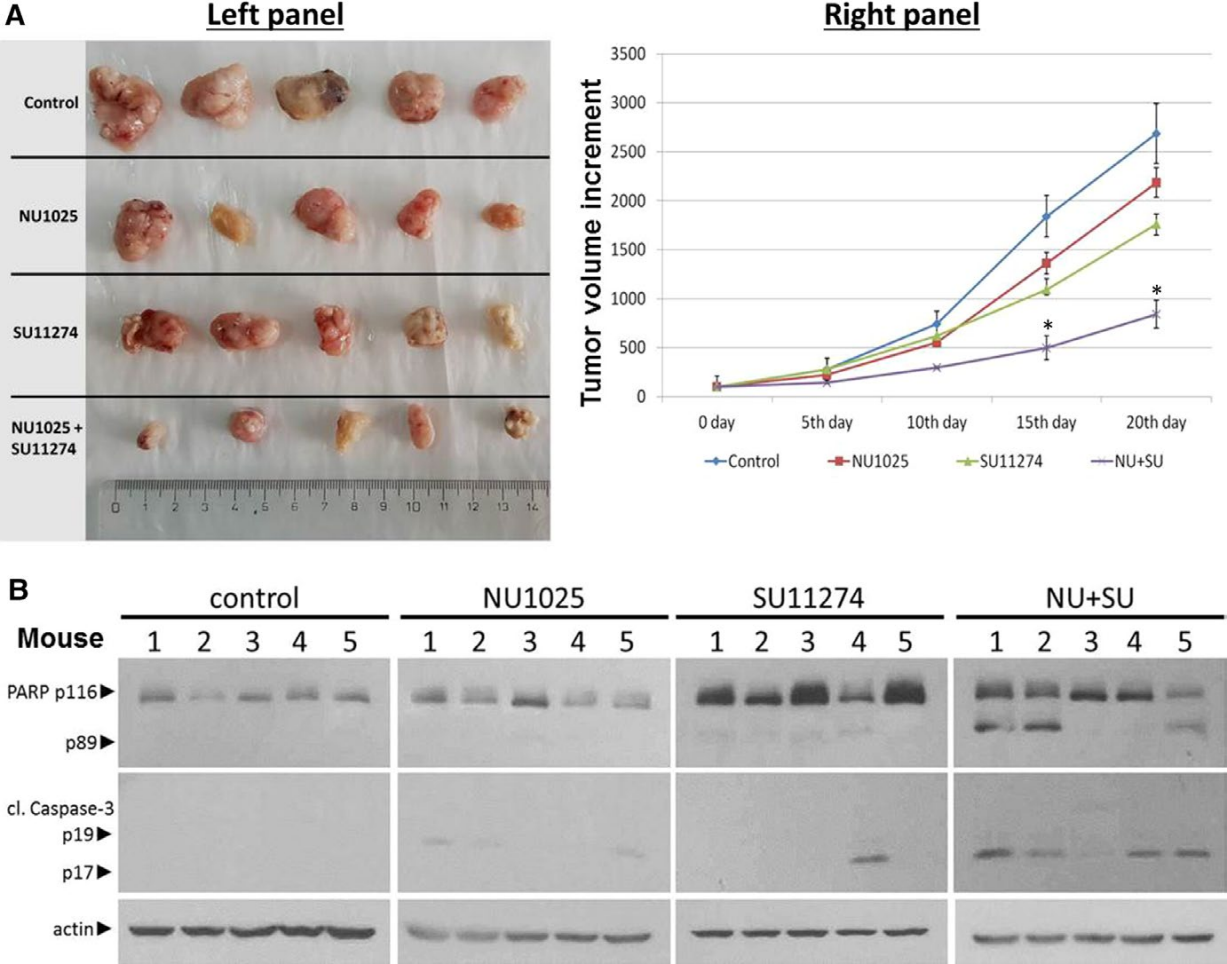
Effect of combinatorial treatment of c‐MET and PARP inhibition in tumour xenograft (AGS) model. AGS cells were inoculated into SCID mice (5 mice per group) on day 0. Mice were inoculated subcutaneously in the right flank with 0.1 mL PBS containing 3 × 106 AGS human gastric cancer cells. When the tumour volume reached ~ 100 mm3, mice were administered with NU1025 (1 mg/mouse) or SU11274 (1 mg/kg), alone or in combination every 4 d for 20 d. Tumour growth was calculated at the indicated time points. Tumour volume was measured 5 d using a calliper and calculated as (width) 2 × length/2. Photographs of excised AGS tumours after receiving different treatments (control, NU1025, SU11274 and NU1025 + SU11274) were captured at the end of 20 d of therapy (A‐left panel) and AGS tumour size progression as a function of time after administration (A‐right panel). Western blot showing the levels of PARP and cleaved caspase‐3 in the subcutaneous tumour tissues isolated from the mice after 20 d of therapy (B)

Collectively of our in vitro experiments, we support the hypothesis that PARP and c‐MET inhibition decrease the viability of GC cell lines. In light of these results, we try to evaluate our in vitro results in xenograft mouse models. For xenograft, we used the AGS cell line, which expressed low BRCA1, BRCA2 and c‐MET compared with HS746T (Figure 1). Thirty days after subcutaneous inoculation of AGS (106 cells/mouse), SCID mice treated intraperitoneally with 1 mg/mouse of SU11274 or NU1025 for 20 days alone or in combinatorial treatment. AGS xenografts were not very sensitive to PARP inhibitor alone (NU1025; Figure 5A). In contrast, SU11274 was slightly more effective in AGS xenograft models. The combinatorial scheme of NU1025 and SU11274 effectively decreases the tumour volume as shown in Figure 5A left panel. Furthermore, the tumour growth curves evaluated that co‐treatment of SU11274 plus NU1025 was more effective in AGS xenograft models compared to either agent alone (Figure 5A right panel).

Following the tumour growth results, the co‐treatment of NU1025 plus SU11274 on AGS xenograft models triggers apoptotic cell death as it was measured through PARP and cleaved caspase‐3 by Western blot analysis after protein extraction from tumours (Figure 5B). The detection of both apoptotic markers in co‐inhibition treatment points (NU1025 and SU11274), evaluates and explains the tumour volume reduction in xenograft models (Figure 5B). Our results highlight the vigorous antitumour activity of PARP and c‐MET inhibition in vitro and in vivo experiments in GC models according to BRCA and c‐MET activity.

## 4 DISCUSSION

In the current study, we support that GC tumours with low levels of BRCA are sensitive to PARP inhibition. Thus, co‐treatment of GC (HS746T and AGS, with higher and lower expression of BRCA1/2 and c‐MET, respectively) cell lines with PARP and c‐MET inhibitors reduces cell viability through apoptosis and attenuated tumour growth in xenograft models. Our results highlight the impact of BRCA1/2 deficiency on gastric tumorigenesis. Moreover, we identified a mechanism that involves γ‐H2AX foci formation, which is well‐known DNA damage marker.^35^, ^36^, ^37^

Over the last few years, a plethora of studies have identified c‐MET as a potential target for GC tumours. Several small molecules have been developed against the c‐MET pathway with promising results in clinical studies.^3^, ^4^ Activation of the c‐MET signalling pathway acts as a tumour protective mechanism against DNA damage.^38^, ^39^ Several studies have investigated the role of c‐MET or PARP‐1 inhibition as putative targets for cancer therapy. In small‐cell lung cancer^40^ and hepatocellular carcinoma (HCC)^41^ cell line models, the combined treatment of the resistant cells with the c‐Met kinase inhibitor SU11274 and other agents significantly hampered cell growth and reversed the increased invasion ability of the cell lines, respectively. Furthermore, SU11274 selectively suppressed the growth of c‐Met overexpressed GC cells.^42^ In breast cancer (BC) model, inhibition of PARP‐1 with NU1025 strongly inhibits BC cell line proliferation with high expression of BRCA1.^43^ Moreover, PARP inhibition (NU1025) enhanced the anti‐proliferative activity and the DNA damage induced by both topoisomerase inhibitors or radiation therapy in glioblastoma cells.^44^

In our experiments, in order to identify the impact of c‐MET in PARP inhibition, we use two different GC cell lines, HS746T and AGS, with high and low protein levels of c‐MET, respectively, in the presence of NU1025 (PARP inhibitor).

The inhibition of PARP did not cause significant inhibition on cell viability of both cell lines. Knockdown of c‐MET with siRNA sensitizes GC cells, especially AGS, in PARP inhibition. These results partially recognize c‐MET as a resistance mechanism to PARP inhibition in the GC cell line model.

Several studies highlight the vital role of BRCA in malignancy. Huang et al^45^ showed that BRCA deficiency sensitizes cancer cells to PARP inhibitors. In the light of this knowledge, we continue with knockdown of BRCA1 and 2 with siRNA in HS726T, a GC cell line with high expression of BRCA1/2 and c‐MET. The silence of BRCA1 or 2 did not change the viability of cells significantly to PARP inhibition. Our data highlight the importance of c‐MET and BRCA1/2 overexpression as a resistance mechanism against PARP inhibitor.

It is well known that c‐MET activation increases the DNA repair function of PARP1.^46^, ^47^ Another study shows that MET inhibition in GC tumours induces the ability of cancer cells to fix DNA damage and increases the effectiveness of the undergoing radiotherapy.^33^ In the CRC model, the combination of crizotinib with mitomycin C (MMC) appeared to synergist and has an anti‐proliferative effect regardless of MSI or BRCA2 status.^48^ According to our findings, we continue with the co‐inhibition of PARP and c‐MET. Our results evaluate the additive effect of co‐administration of NU1074 plus SU11274 in GC cells. This combinatorial treatment reduces clonogenic activity in HS746T and cell viability through apoptotic cell death. Moreover, we also observed a significant increase in DNA damage in both GC cell lines. In more details, after the silence of BRCA1/2 in HS746T GC cell line, co‐administration of NU1025 plus SU11274 sharply increased the DNA damages as it was evaluated through the induction of γ‐Η2ΑΧ. Furthermore, our results are in line with already published data^49^ shows that this combinatorial approach effectively decreases basic cellular properties like cell proliferation and clonogenicity, and on the other hand, induces apoptotic cell death.

Du et al^31^ studied the role of c‐MET and PARP co‐administered in triple‐negative cancer xenograft mouse models and observed better outcomes than separate administration. Besides, the same study supports that the NSCLC cell line H1993 with high expression of c‐MET may have benefited from this combination therapy.^31^ With our results, we support the impact of co‐treatment of SU11274 and NU1025 on in vitro and in vivo experiments. For in vivo experiments, we used the AGS cell line because of the low expression of BRCA1/2 and c‐MET. In fact, in AGS xenograft tumour models, co‐inhibition of PARP and c‐MET significantly reduced tumour growth compared to control or inhibitor alone.

PARP1 is a crucial protein for sensing single‐stranded (ss)DNA breaks (SSBs).^50^ Thus, several agents are developed in order to inhibit PARP (PARPi) activation. PARPi not only inhibits the activity of PARP1 but also traps the protein on the damaged DNA. Such protein‐DNA structures add to the cytotoxic effect of different drugs by block the formation of replisome.^51^, ^52^ Cells require functional homologs recombination (HR) mechanism to resolve these damages and resume cell‐cycle progression and PARPi appears to induce cell death in HR‐deficient tumours.^53^ Loss of PARP1 expression was not tolerated in cancer cells carrying a mutation in the BRCA1 gene. Besides, different mutations in BRCA1 not only tolerate PARP1 loss but can also become PARPi resistant due to mutations in PARP1 that lead to protein loss or decreased DNA trapping.^53^ Consequently, the combination of PARP and c‐MET inhibitors may be a putative and more effective chemotherapeutic scheme for GC patients.

It is known that BRCA deficiency causes problems in the DNA damage repair mechanism. Tumours with mutant BRCA genes are more sensitive to PARP inhibition.^22^, ^54^ Therefore, it is interesting to consider whether dual inhibition of PARP and c‐MET using NU1074 and SU11274 inhibitor, respectively, reveals a proficient beneficial effect for BRCA deficiency gastric malignancies. For this purpose, we used GC cell lines with high levels (Hs746T) and low levels of c‐MET (AGS). We evaluated the molecular mechanisms that contribute to the resistance of the PARP inhibition in the GC cell line model through the inhibition of c‐MET and silence of BRCA1/2. We observed a further reduction in cell viability through apoptotic cell death and increasing levels of γ‐H2AX in PARP or c‐MET inhibition separately and in combination. Moreover, in AGS xenograft mouse model, co‐administration of NU1074 and SU11274 was even more efficient compared to c‐MET or PARP inhibitors alone.

An assay for histone γH2AX generally reflects the presence of double‐strand breaks in DNA. In our experiments, the detection of cl. Caspase‐3 and PARP in co‐inhibition of PARP and c‐MET does not correlate with the protein levels of γ‐H2AX by Western blot analysis.^55^ However, it should be pointed out that Western blotting analysis cannot differentiate apoptotic than DDB γ‐H2AX, whereas immunofluorescence microscopy does. With confocal microscopy, we detected increased levels of γ‐H2AX around the nucleus in a ring‐shape formation. The ring constitutes an epigenetic landmark of early apoptosis. It differs from the focal patterns of DNA damage foci produced by DNA damaging agents.^56^ This highlights the correlation of γ‐H2AX with the increasing levels of apoptotic markers in the co‐inhibition of PARP and c‐MET in GC in vitro experiments. Many studies have already identified abnormal and increased c‐Met activation in gastric malignancies,^57^ probably due to the positive correlation between c‐Met and resistance to PARP inhibition.^58^ It is noteworthy that BRCA defiance's existence appears to be positively linked with improved survival and increased sensitivity to chemotherapy of tumours.^53^ Based on our results, GC patients with high expression of c‐Met may have benefited from a co‐inhibition c‐Met and PARP therapy, reducing the deficiency in repairing the DNA and consequently diminishing the tumour size. In addition to GC patients, with low expression of BRCA1/2, treatment with PARP inhibitors will be beneficial. Thus, the additive effect of PARP and c‐MET represents a putative therapeutic strategy for GC patients with BRCA deficiency status.

There were some limitations to this study. First, the sample size of in vivo experiments may not have been large enough. Second, an in‐depth analysis of signalling pathways that are controlled by c‐MET has not been tested. Third, the relationship between BRCA1/2 and c‐MET needs further tests; for example, mutations on BCA1/2 and c‐MET can change the effect of NU1025 and SU11274 inhibitors. Nevertheless, these limitations may point to the future direction along the lines of this study.

## 5 CONCLUSION

Our results collectively evaluate the additive effect of c‐MET and PARP inhibition in the GC cell line model. Both inhibitors (NU1025 and SU11274) trigger DNA damage response and apoptotic cell death, resulting in the reduction of cell viability of GC cell lines, especially with BRCA deficiency status. Furthermore, the xenograft mouse model supports our hypothesis that NU1074 plus SU11274 displays a significant reduction of tumour growth in a cell line with low levels of BRCA1/2 and c‐MET. Our results evaluate that the co‐administration of NU1074 and SU11274 has an additive effect. Based on these findings, further clinical testing of this combinatorial scheme is suggested in patients with locally advanced and/or metastatic gastric cancer.

## CONFLICTS OF INTEREST

The authors declare no potential conflicts of interest.

## AUTHOR CONTRIBUTION

**Evangelos Koustas:** Conceptualization (equal); Data curation (equal); Formal analysis (equal); Investigation (equal); Methodology (equal). **Michalis V. Karamouzis:** Conceptualization (equal); Data curation (equal); Formal analysis (equal). **Panagiotis Sarantis:** Data curation (supporting); Investigation (supporting); Methodology (supporting). **Dimitrios Schizas:** Data curation (supporting); Formal analysis (supporting). **Athanasios G Papavassiliou:** Conceptualization (equal); Data curation (equal); Formal analysis (equal).

## INSTITUTIONAL ANIMAL CARE AND USE COMMITTEE STATEMENT

All procedures were carried out in accordance with the guidelines for animal experimentation of the National and Kapodistrian University of Athens, Medical School Bioethics Committee in agreement with the European Union (approval no. 3233/26‐06‐2018).

## ETHICS APPROVAL AND CONSENT TO PARTICIPATE

All authors confirm that any aspect of the work covered in this manuscript that has involved either cell lines or animal models has been conducted with the ethical approval of all relevant bodies and that such approvals are acknowledged within the manuscript.

## CONSENT FOR PUBLICATION

All authors consent for the publication of the manuscript.

## Supporting information

Figure S1

## Data Availability

All authors declare that the data are available upon request.
