# Supplementary figures and images for: Inhibition of c‐MET increases the antitumour activity of PARP inhibitors in gastric cancer models
Source: J Cell Mol Med. 2020 Jul 20;24(18):10420–31. doi: 10.1111/jcmm.15655 (PMC7521333; doi:10.1111/jcmm.15655)

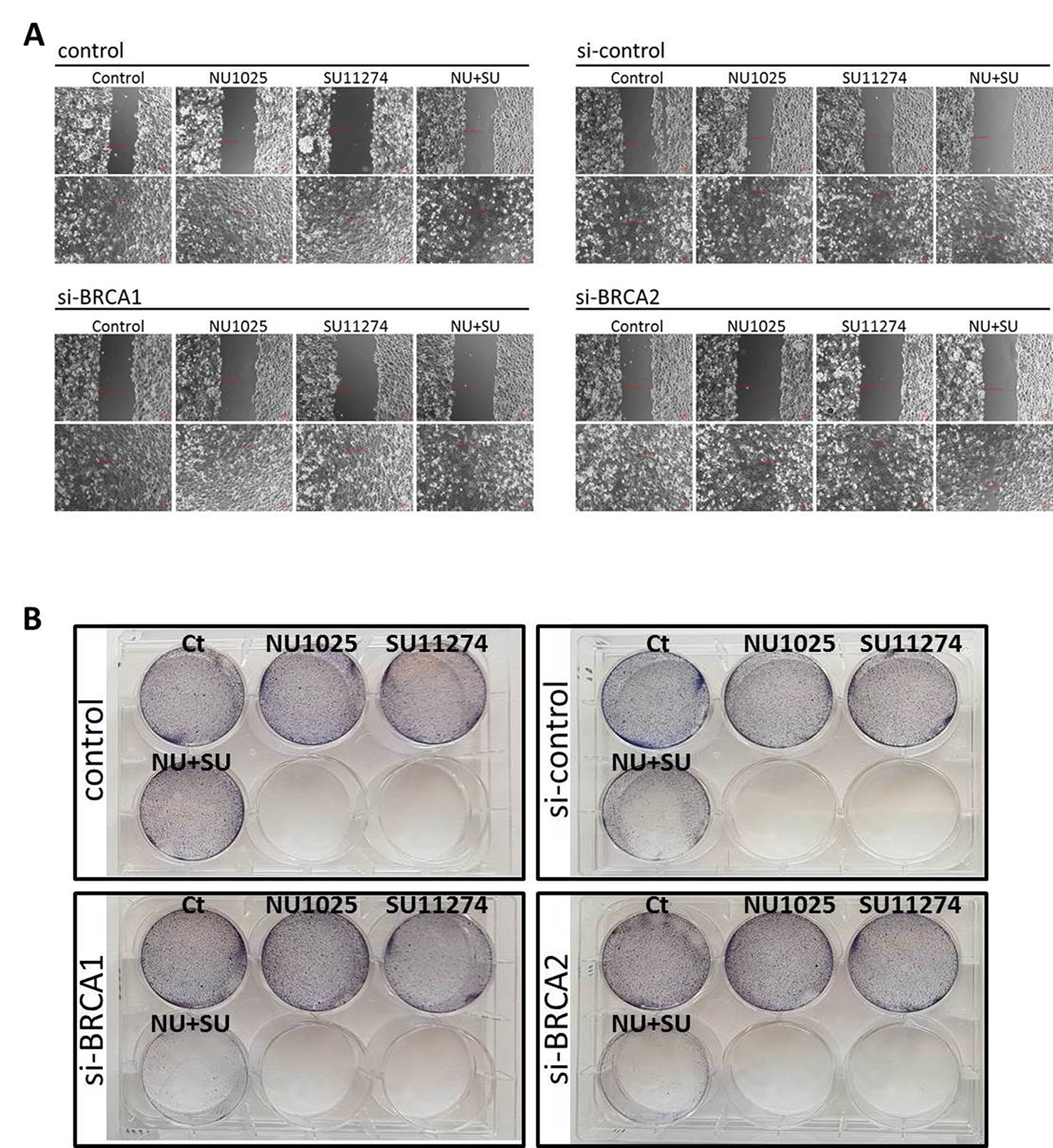

Supplement: Supplementary file 1 — Figure S1 [file JCMM-24-10420-s001.tif]
